# Synthesis and optical properties of pyrrolidinyl peptide nucleic acid carrying a clicked Nile red label

**DOI:** 10.3762/bjoc.10.224

**Published:** 2014-09-11

**Authors:** Nattawut Yotapan, Chayan Charoenpakdee, Pawinee Wathanathavorn, Boonsong Ditmangklo, Hans-Achim Wagenknecht, Tirayut Vilaivan

**Affiliations:** 1 Organic Synthesis Research Unit, Department of Chemistry, Faculty of Science, Chulalongkorn University, Phayathai Road, Patumwan, Bangkok 10330, Thailand; 2 Institute of Organic Chemistry, Karlsruhe Institute of Technology (KIT), Fritz-Haber-Weg 6, 76131 Karlsruhe, Germany

**Keywords:** click chemistry, deoxyribonucleic acid, DNA bulge, fluorescence, nucleic acids, solvatochromism

## Abstract

DNA or its analogues with an environment-sensitive fluorescent label are potentially useful as a probe for studying the structure and dynamics of nucleic acids. In this work, pyrrolidinyl peptide nucleic acid (acpcPNA) was labeled at its backbone with Nile red, a solvatochromic benzophenoxazine dye, by means of click chemistry. The optical properties of the Nile red-labeled acpcPNA were investigated by UV–vis and fluorescence spectroscopy in the absence and in the presence of DNA. In contrast to the usual quenching observed in Nile red-labeled DNA, the hybridization with DNA resulted in blue shifting and an enhanced fluorescence regardless of the neighboring bases. More pronounced blue shifts and fluorescence enhancements were observed when the DNA target carried a base insertion in close proximity to the Nile red label. The results indicate that the Nile red label is located in a more hydrophobic environment in acpcPNA–DNA duplexes than in the single-stranded acpcPNA. The different fluorescence properties of the acpcPNA hybrids of complementary DNA and DNA carrying a base insertion are suggestive of different interactions between the Nile red label and the duplexes.

## Introduction

Fluorescent labels are important tools for investigating the structure and dynamics of biomolecular interactions [1–3]. Traditionally, the biological macromolecules are labeled with two or more dyes which can interact in a conformation/distant-dependent manner via Förster resonance energy transfer (FRET) [4–6]. Alternatively, the FRET pairs can be replaced by an environmentally sensitive label that can change its fluorescence in response to its altered micro-environment [7–9]. Nile red is a member of the benzophenoxazine dye family which exhibits several interesting features including a high photostability, high fluorescence quantum yield, broad working pH range, long excitation and emission wavelengths, and solvatochromic properties [10]. Applications of Nile red as a staining dye in histology [11–12] and as a probe for the sensing of polarity and hydrophobicity [13] are well-known. Nile red has been used as a DNA label, either as a base modifier [14–16], a base replacement [17] or a backbone-tethered label [18–19]. However, in most cases the formation of DNA duplexes does not yield significant fluorescence changes in the Nile red, unless it is used in combination with another dye such as pyrene to form an energy transfer pair [20–21]. A related phenoxazine dye – Nile blue – has also been incorporated into DNA as a base replacement, again without showing significant structure-induced fluorescence change [22].

Peptide nucleic acid or PNA is an electrostatically neutral analogue of DNA which can form very stable duplexes with DNA and RNA in a highly sequence specific fashion. PNA–DNA duplexes have different structural morphology and electrostatic potential surfaces from DNA–DNA duplexes and therefore they are interacting differently with DNA-binding dyes [23–25]. We had recently introduced a new conformationally constrained pyrrolidinyl PNA known as acpcPNA that shows several unique properties [26–28]. Its potential applications as a probe for DNA sensing are well-established. For example, we have developed singly labeled acpcPNA probes that can give a fluorescence change in response to hybridization to DNA [29–31]. Due to its solvatochromic properties, Nile red is a potential candidate to be used in combination with PNA to develop a hybridization probe which not only differentiates between complementary and non-complementary DNA, but can also report on local structural variations. Except for an example from our group on the conformation control of Nile red-labeled DNA by acpcPNA [32], no combination of the Nile red label and PNA has yet been reported in the literature. It is therefore our purpose to develop a facile method for synthesizing Nile red-labeled acpcPNA based on the click strategy [33–36] and to investigate its optical properties.

## Results and Discussion

**Scheme 1:**
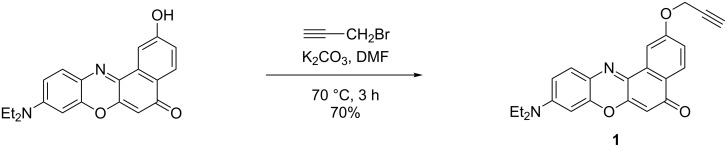
Synthesis of propargylated Nile red **1**.

At least two different means to introduce the Nile red label onto DNA by using click chemistry have been reported in the literature. One involves the clicking of in situ generated 5-azidodeoxyuridine-containing DNA with propargyl Nile red [16], the other employs DNA bearing 2’-propargylated nucleotides and azide-modified Nile red [19]. An approach related to the latter was used in this work, although the azide function was placed on the PNA instead of the Nile red, and the clicking was carried out on the solid support rather than in solution phase. The propargylated Nile red label **1** was synthesized in 70% yield by alkylation of the known 2-hydroxy Nile red [37–38] with propargyl bromide in the presence of K_2_CO_3_ in DMF (Scheme 1).

**Scheme 2:**
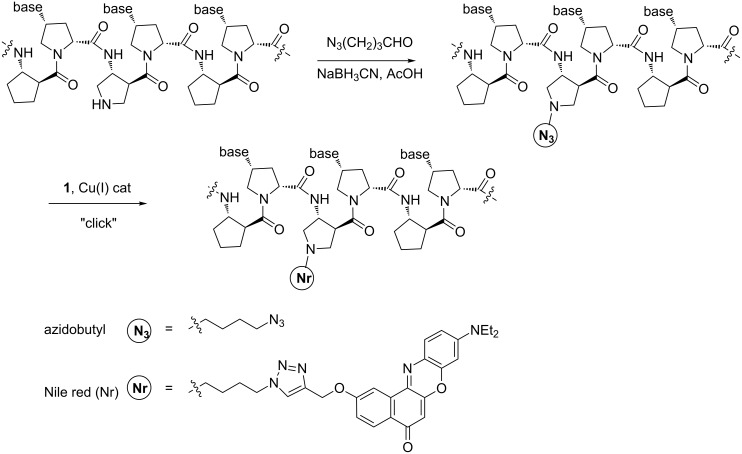
Synthesis of azidobutyl- and Nile red-modified acpcPNA.

**Figure 1:**
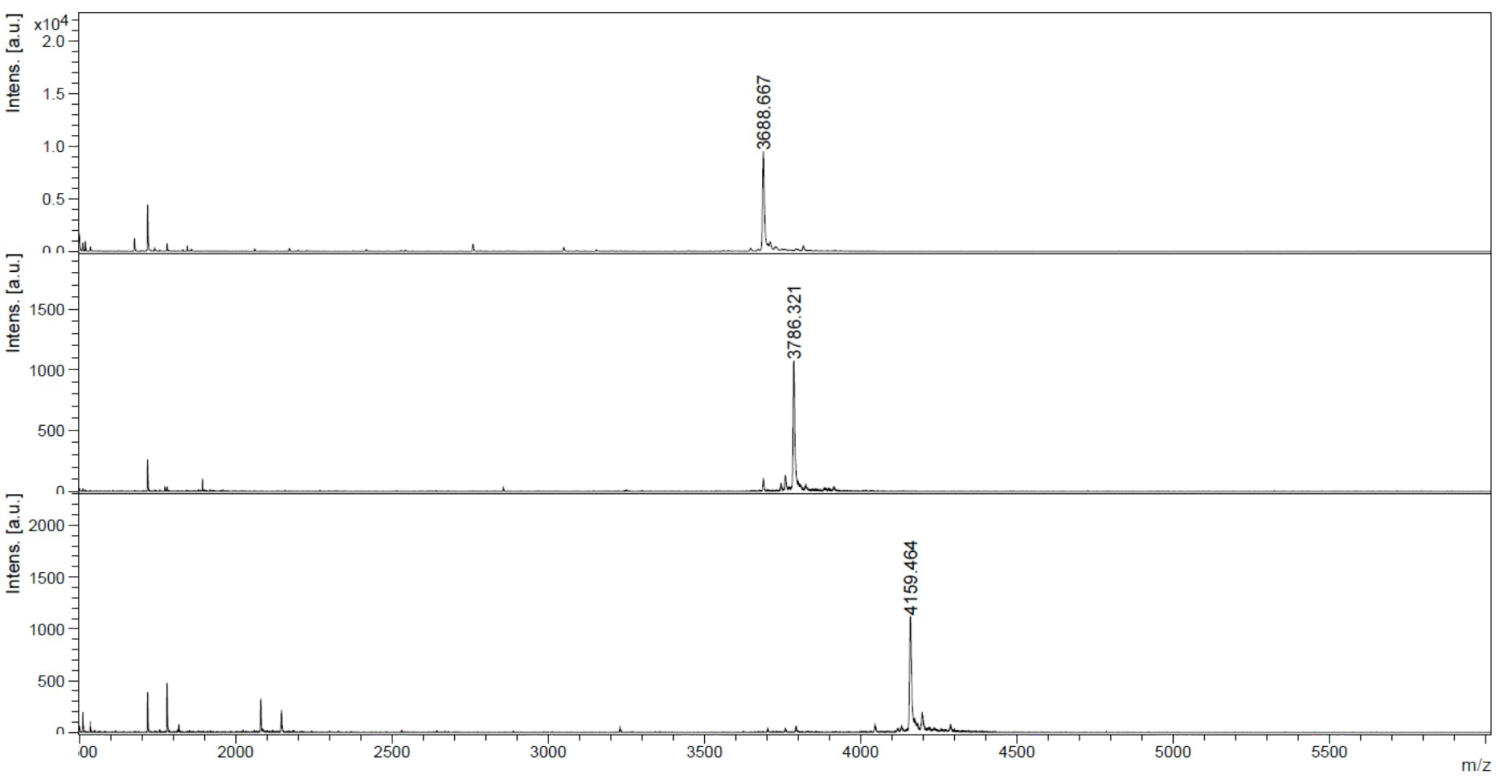
MALDI–TOF mass spectra of the crude 10mer acpcPNA before (top) (calcd *m*/*z* 3688.0), and after functionalizing with the azidobutyl group (middle) (calcd *m*/*z* 3785.1), and followed by clicking with Nile red (bottom) (calcd *m*/*z* 4157.5).

The alkyne-containing compound **1** was clicked onto the backbone of acpcPNA that had been pre-functionalized with an azidobutyl group by a reductive alkylation strategy previously reported by our group (Scheme 2) [31]. Two lysine residues were incorporated at the N- and C-termini of the acpcPNA to ensure a sufficient solubility in aqueous solution. Five acpcPNA sequences, each of which singly labeled at the backbone with Nile red under different sequence context, were successfully synthesized and characterized by MALDI–TOF MS (Figure 1 and Table 1). Isolated yields in the range of 6–18% were obtained (0.5 μmol scale), which are typical for solid phase synthesis, whereby the majority of material loss occurred during HPLC purification. All Nile red-labeled acpcPNAs are freely soluble in water (>1 mM), providing a bright blue solution.

**Table 1:** Sequence, isolated yield and characterization data of Nile red-labeled acpcPNA.

PNA	sequence (N to C)^a^	*t*_R_ (min)^b^	% yield^c^	*m*/*z*^d^ (calcd)	*m*/*z*^e^ (found)

**10mer-Nr**	GTAGA(Nr)TCACT	33.6	6.3	4157.5	4155.1
**11merAA-Nr**	CATAA(Nr)AATACG	34.2	18.5	4491.9	4491.1
**11merCC-Nr**	CATAC(Nr)CATACG	34.6	11.4	4443.9	4441.8
**11merGG-Nr**	CATAG(Nr)GATACG	32.8	15.2	4523.9	4523.2
**11merTT-Nr**	CATAT(Nr)TATACG	34.3	8.4	4473.9	4473.0

^a^All sequences were end-capped at N- and C-termini with *N*-acetyl-L-lysine and L-lysinamide, respectively. ^b^HPLC conditions: C18 column 4.6 × 50 mm, 3 µ, gradient 0.1% TFA in H_2_O:MeOH 90:10 for 5 min then linear gradient to 10:90 over 30 min, flow rate 0.5 mL/min, 260 nm. ^c^Isolated yield (determined spectrophotometrically) after HPLC purification. ^d^Average mass of [M + H]^+^. ^e^MALDI–TOF.

**Figure 2:**
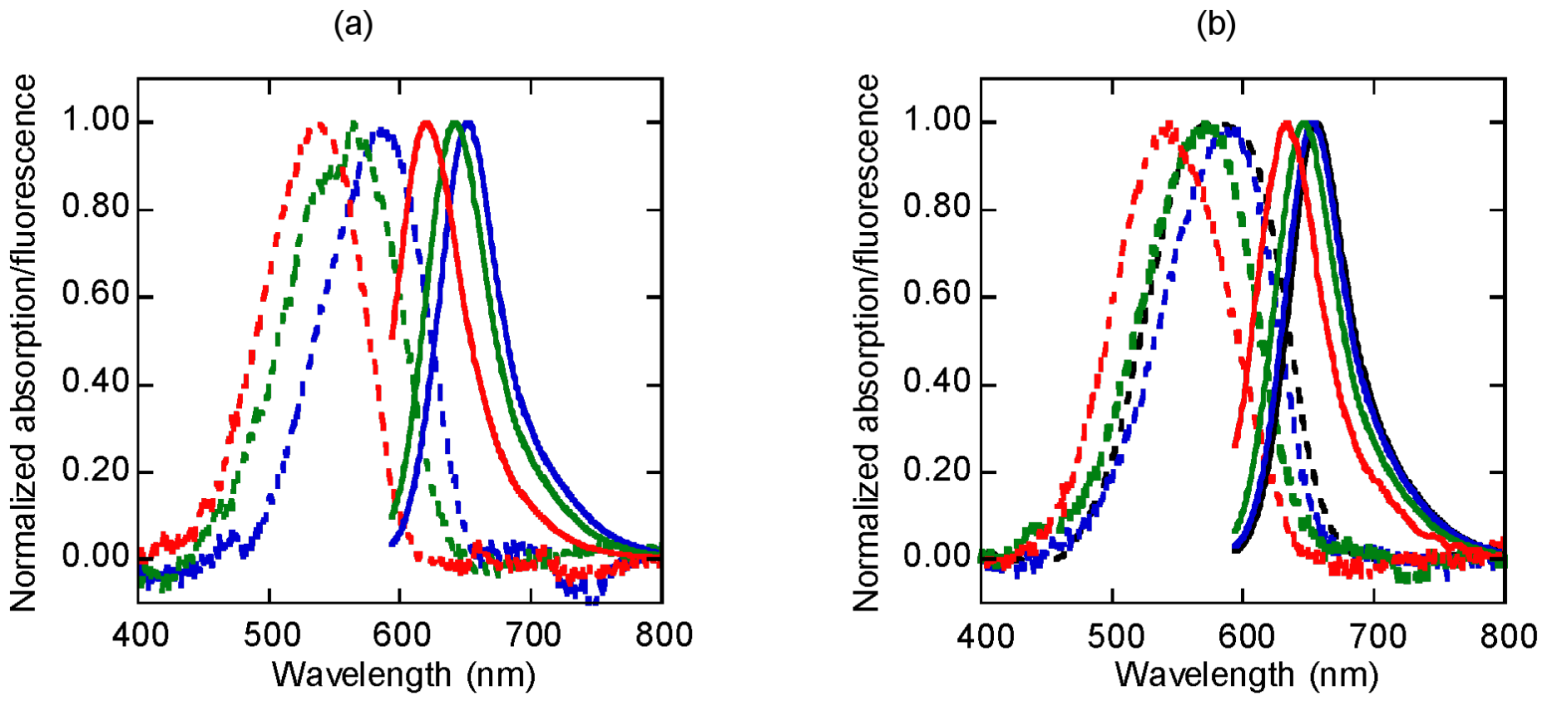
Normalized absorption (---) and fluorescence (––) spectra of (a) propargyl Nile red **1** and (b) Nile red-labeled acpcPNA **10mer-Nr** in 10 mM sodium phosphate buffer (pH 7.0):acetonitrile: black = 100:0; blue = 80:20; green = 50:50; red = 0:100. All measurements were carried out in 10 mM sodium phosphate buffer (pH 7.0), [PNA] and [**1**] = 1.0 µM at 20 °C with λ_ex_ = 580 nm.

The UV–vis spectrum of the free propargyl Nile red label **1** in acetonitrile (Figure 2a) showed absorption and fluorescence emission maxima at 538 nm and 620 nm, with a fluorescence quantum yield (Φ_F_) of 0.65, which is quite comparable to other Nile red derivatives reported in the literature [10]. As the polarity of the solvent increases with the addition of aqueous phosphate buffer, both the absorption and the fluorescence maxima shifted to longer wavelengths with a concomitant decrease in the fluorescence quantum yields (50% MeCN: λ_abs_ 564 nm, λ_em_ 641 nm, Φ_F_ 0.29; 20% MeCN: λ_abs_ 587 nm, λ_em_ 651 nm; Φ_F_ 0.14). In aqueous phosphate buffer (10 mM, pH 7.0), the Nile red-labeled acpcPNA **10mer-Nr** exhibited a broad absorption peak centered at 575 nm and a fluorescence emission at 656 nm, respectively (Figure 2b). The solvatochromic property of the Nile red-labeled acpcPNA is demonstrated as shown by the progressively blue-shifted absorption and fluorescence emission maxima in the presence of acetonitrile (100% MeCN: λ_abs_ 544 nm, λ_em_ 634 nm; 50% MeCN: λ_abs_ 569 nm, λ_em_ 647 nm; 20% MeCN: λ_abs_ 592 nm, λ_em_ 654 nm). The fluorescence spectrum of the Nile red-labeled acpcPNA is notably red-shifted compared to the fluorescence spectrum of the free Nile red label in the same solvent. This suggests possible interactions between the Nile red label and the PNA base or backbone. Nevertheless, the Nile red in the acpcPNA **10mer-Nr** was not quenched as indicated by the quantum yield value being similar to that of the free label (**10mer-Nr**: Φ_F_ = 0.16; **1**: Φ_F_ = 0.14 in 20% MeCN). Unfortunately, the value can only be compared in an acetonitrile:water mixture as compound **1** is essentially insoluble in water.

**Figure 3:**
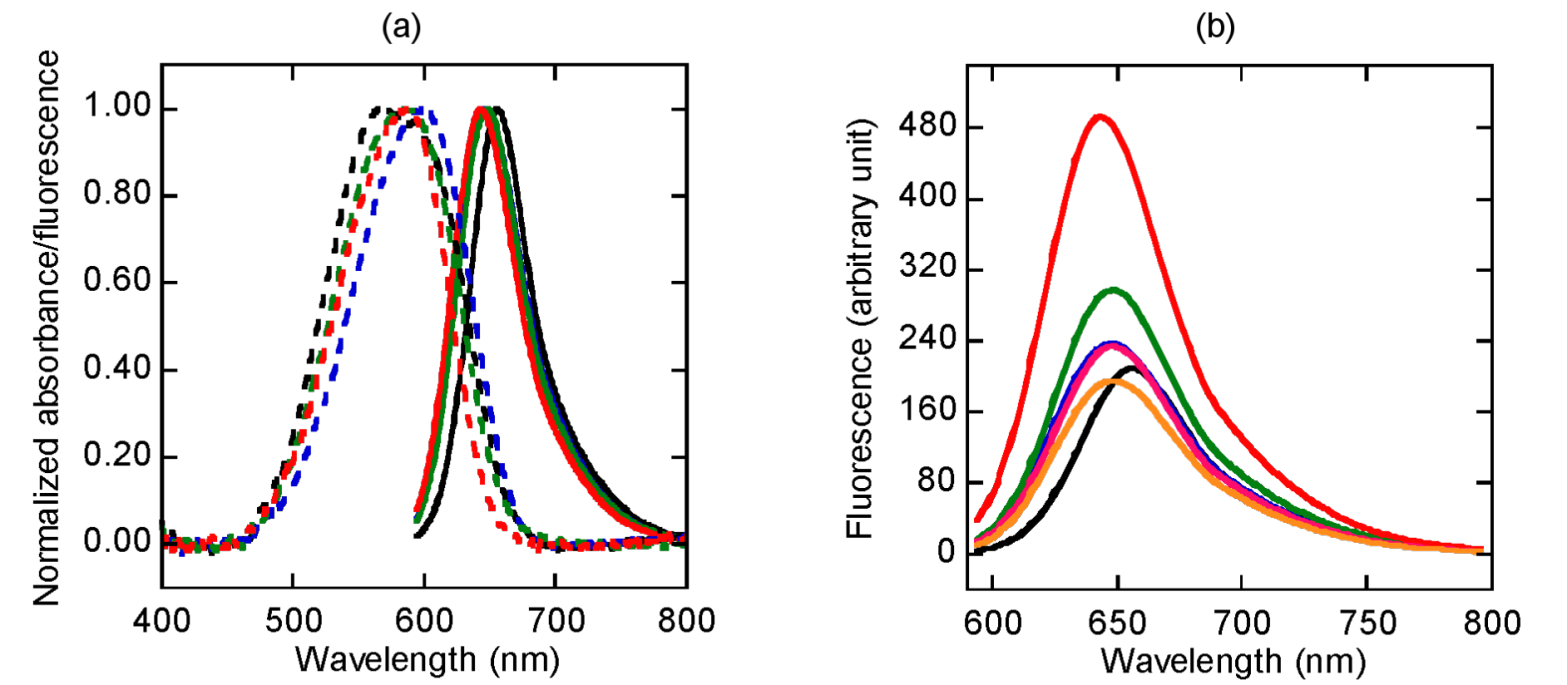
(a) Normalized absorption (---) and fluorescence (––) spectra and (b) fluorescence spectra of Nile red-labeled acpcPNA **10mer-Nr** in the absence (black) and presence of various DNA sequences (5’→3’): blue = AGTGATCTAC (complementary); green = AGTGCTCTAC (mismatched); red = AGTGA**C**TCTAC (bulged); pink = AGTGC**C**TCTAC (bulged with mismatch); orange = AGT**C**GATCTAC (misplaced bulge). All measurements were carried out in 10 mM sodium phosphate buffer (pH 7.0), [PNA] = 1.0 µM; [DNA] = 1.2 µM at 20 °C with λ_ex_ = 580 nm.

Melting temperature data and optical properties of the Nile red-labeled acpcPNAs and their hybrids with various DNA are summarized in Table 2. Thermal denaturation experiments suggest that the Nile red-labeled acpcPNA **10mer-Nr** can form a stable hybrid with complementary DNA. In contrast to some other labels such as pyrene which usually destabilize acpcPNA–DNA duplexes [30–31], the *T*_m_ of the complementary DNA hybrid of Nile red-labeled PNA (58.8 °C by UV or 56.9 °C by fluorescence) was comparable to the *T*_m_ of unlabeled acpcPNA with an identical sequence (57.6 °C) [26]. Absorption spectra of **10mer-Nr** and its complementary DNA duplex show differences in the Nile red region as shown by the sharpening and red shift of the absorption maxima (λ_max_ = 575 and 598 nm in single stranded and duplex, respectively). Importantly, fluorescence spectra of the duplex showed a pronounced blue shift of the emission maxima (647 nm) compared to the single-stranded PNA (656 nm) as well as a small fluorescence increase (1.36 fold at 643 nm) (Figure 3a,b). The blue-shifted fluorescence maxima and increased quantum yields of Nile red suggest that the Nile red chromophore in complementary PNA–DNA duplexes was placed in a less polar environment compared to its placement in single-stranded PNA. Although the related benzophenoxazine dye Nile blue binds to DNA by intercalation, the binding results in a bathochromic shifting and a quenched fluorescence [39–40]. Based on this, together with the fact that PNA–DNA duplexes are not good substrates for intercalative binding [23], we propose that the Nile red is more likely to bind to the groove of the PNA–DNA duplex rather than intercalate into the base stacks.

**Table 2:** *T*_m_ and optical properties of Nile red-labeled acpcPNA^a^.

PNA	DNA (5′ to 3′)^b^	*T*_m_ (°C)^c^	λ_abs_	λ_em_^d^	Φ_F_^e^	*F*/*F*_0_^f^

**10mer-Nr**	none	–	575	656	0.11	–
	AGTGATCTAC	58.8 (56.9)	598	647	0.15	1.36
	AGTGCTCTAC	37.5 (36.7)	585	648	0.17	1.72
	AGTCATCTAC	37.7 (37.5)	589	649	0.14	1.43
	AGTGACCTAC	40.8 (41.3)	592	652	0.15	1.70
	AGTGA**C**TCTAC	46.9 (52.0)	589	643	0.29	2.91
	AGTGA**A**TCTAC	40.1 (46.7)	588	648	0.20	2.60
	AGTGA**T**TCTAC	54.7 (52.7)	591	649	0.22	2.65
	AGTGA**G**TCTAC	42.2 (46.0)	593	645	0.20	3.31
	AGT**C**GATCTAC	N.D.^g^	591	648	0.14	1.16
	AGTGATC**C**TAC	N.D.^g^	597	653	0.10	0.87
	AGTGC**C**TCTAC	N.D.^g^	585	648	0.17	1.45
	AGTGA**C**CCTAC	N.D.^g^	593	652	0.13	1.23
	AGTGA**C**TCCAC	N.D.^g^	585	647	0.13	1.24
**11merAA-Nr**	none	–	598	657	0.15	–
	CGTATTTTATG	76.0	600	651	0.23	1.55
	CGTATT**C**TTATG	74.7	593	645	0.33	2.34
**11merCC-Nr**	none	–	594	656	0.08	–
	CGTATGGTATG	67.4	594	646	0.19	2.49
	CGTATG**C**GTATG	71.2	598	644	0.13	1.79
**11merGG-Nr**	none	–	599	660	0.04	–
	CGTATCCTATG	(62.3)	599	655	0.15	3.27
	CGTATC**C**CTATG	(62.3)	599	652	0.10	2.46
**11merTT-Nr**	none	–	588	655	0.09	–
	CGTATAATATG	76.6	602	654	0.19	2.05
	CGTATA**C**ATATG	76.0	590	646	0.29	3.44

^a^All measurements were carried out in 10 mM sodium phosphate buffer (pH 7.0), [PNA] = 1.0 µM; [DNA] = 1.2 µM at 20 °C. ^b^Underlined and boldface letters in DNA sequences indicate the position of mismatch and base insertion, respectively. ^c^Determined by UV–vis (260 nm) and/or fluorescence spectrophotometry (643 nm, shown in parentheses). ^d^λ_ex_ = 580 nm. ^e^Cresyl violet was used as a standard (Φ = 0.54 in MeOH) [41]. ^f^*F*/*F*_0_ was calculated from the ratio of fluorescence of duplex divided by the single-stranded PNA at 643 nm. ^g^Not determined.

In the presence of a mismatched base in the DNA strand, the *T*_m_ of the duplex was decreased as expected. For example, the mismatched hybrid of **10mer-Nr** with the DNA carrying a mismatched base in close proximity to the position of the Nile red label showed *T*_m_ values in the range of 37–41 °C. Fluorescence spectra of these mismatched DNA duplexes showed blue-shifted and enhanced fluorescence emissions similar to the complementary duplex, which ranged from 1.4 to 1.7 fold compared to the one of single-stranded Nile red-labeled acpcPNA. This suggests that the Nile red label can still noticeably interact with the mismatched duplexes.

The most remarkable feature of the Nile red-labeled acpcPNA **10mer-Nr** is the relatively large fluorescence increase after the hybridization with DNA targets which carry a base insertion in close proximity to the Nile red label. As shown in Table 2, these duplexes showed more pronounced fluorescence increases (2.6–3.3 fold) than the complementary or mismatched duplexes (1.4–1.7 fold relative to the single-stranded **10mer-Nr**). The fluorescence maxima were also further blue-shifted relative to the single-stranded and complementary duplexes of Nile red-labeled acpcPNA (Figure 3a,b). These results suggest that the Nile red label in these duplexes adopts a different configuration to the complementary and the single-mismatched duplexes. When the DNA strand carries an extra inserted base, the only way it can form a stable hybrid is to form a bulge on the DNA strand at the insertion site. This is supported by the unusually high *T*_m_ values (46–53 °C) for these duplexes. In addition, consistent fluorescence increases were observed regardless of the nature of the inserted base. Based on these findings we propose that the extra DNA base is looped out to form a bulge which can accommodate the Nile red label. A similar binding mode has been proposed earlier for pyrene-labeled DNA [42]. When the inserted base was misplaced, i.e., away from the Nile red label, or when a mismatch base was introduced elsewhere in the DNA strand, the fluorescence change was small (Figure 3b, Figure S11 and Figure S12, Supporting Information File 1), suggesting that the fluorescence increase was due to a specific interaction between the Nile red label and the bulge site. The addition of β-cyclodextrin to the bulged duplexes caused no change in the fluorescence of the Nile red label, whereas a marked blue shift was observed with single-stranded PNA (8 nm) (Figure S17, Supporting Information File 1). This experiment confirmed that the Nile red label is buried well within the hydrophobic pocket of the bulged duplexes and therefore not available to form an inclusion complex with the cyclodextrins [17]. Less pronounced shifts were observed with complementary and mismatched duplexes (2 and 4 nm, respectively) upon the addition of cyclodextrin, which indicates possible interactions between the groove-bound Nile red and cyclodextrin.

To better understand the effect of neighboring bases on the optical properties of the Nile red-labeled acpcPNA and its duplexes with DNA, four Nile red-labeled acpcPNA sequences (**11merAA-Nr**, **11merCC-Nr**, **11merGG-Nr**, **11merTT-Nr**) were synthesized. These four Nile red-labeled acpcPNA sequence exhibited differences only at the bases flanking the Nile red label, For single-stranded PNA, the order of fluorescence quantum yields was AA > TT ~ CC > GG. This suggests a more efficient quenching of the Nile red label by neighboring G than by other nucleobases. A is almost non-quenching as shown by the high quantum yield of **11merAA-Nr** (Φ_F_ = 0.15), which is comparable to the high quantum yield of the free Nile red label (Φ_F_ = 0.14). On the other hand, the presence of two flanking G in **11merGG-Nr** resulted in a decrease of the quantum yield of more than 70% (Φ_F_ = 0.04). Upon hybridization with complementary DNA targets, the fluorescence quantum yields of all four hybrids were increased to a similar range (Φ_F_ = 0.15–0.23). However, since the initial fluorescence of **11merGG-Nr** was low, the fluorescence change was more pronounced (3.27 fold) than other sequences (1.55–2.49 fold). When a bulge was introduced, a larger fluorescence increase was observed in duplexes with neighboring A-T than G-C pairs. Accordingly, the bulged duplexes of **11merAA-Nr** and **11merTT-Nr** became more fluorescent than the corresponding complementary duplexes. Opposite results were observed with **11merCC-Nr** and **11merGG-Nr**, that is, the complementary duplexes exhibited higher fluorescence than the bulged duplexes. In all cases the fluorescence emission maxima of the bulged duplex were at shorter wavelengths than fluorescence emission maxima of the complementary duplexes and single-stranded PNA. These results clearly support our hypothesis of the different placement of the Nile red label in the complementary and bulged duplexes.

Although Nile red has been previously incorporated in DNA either through a base substitution or modification, these Nile red-labeled DNAs do not show appreciable fluorescence changes in response to the hybridization with DNA regardless of the mode of Nile red attachment [14,17–18]. In most cases, the pairing of Nile red-labeled DNA with another DNA strand resulted in unchanged or decreased fluorescence quantum yields. This, together with the red-shifted absorption and fluorescence spectra in comparison to single-stranded DNA, suggests that Nile red may intercalate into the base stack of DNA–DNA duplexes. The behavior of the Nile red label in acpcPNA is completely different, as shown by the consistent increase in the fluorescence quantum yield upon hybridization with complementary DNA irrespective of the nature of the flanking bases. In such duplexes, the Nile red is expected to interact with the PNA–DNA duplexes by means of groove binding, which results in a lower localized polarity around the Nile red chromophore and gives rise to the observed blue shifts and the fluorescence increase. In addition, a larger increase in fluorescence was also observed with DNA targets that can form a bulge in the vicinity of the Nile red label. We propose that the Nile red label is buried within the looped out structure of the bulged duplex, which gives rise to an even more pronounced blue shift and fluorescence increase, except when there are nearby G-C base pairs which may quench the fluorescence.

## Conclusion

We successfully synthesized the propargylated Nile red **1** and clicked it onto acpcPNA with an azide-modified backbone. The solvatochromic properties of the Nile red label is retained in the labeled acpcPNA. Quenching of the Nile red label by neighboring bases in acpcPNA increases in the order of A > T ~ C > G. The hybridization with fully complementary DNA and DNA with an inserted base consistently resulted in blue-shifted and enhanced fluorescence. This indicates that the Nile red label in acpcPNA–DNA duplexes is in a more hydrophobic environment compared to when the Nile red label is in single-stranded acpcPNA. Based on spectroscopic evidence, we propose that either the Nile red label resides within the groove (complementary duplexes) or the hydrophobic pocket formed by the looped-out base (bulged duplexes) rather than intercalating into the base stacks.

## Experimental

### General remarks

All chemicals were obtained from standard suppliers and used as received. Anhydrous DMF for peptide synthesis was purchased from RCI Labscan (Thailand). All other solvents were AR or HPLC grade and were used without further purification. Oligonucleotides were obtained from Pacific Science (Thailand) or BioDesign (Thailand). The water used in all experiments was obtained from an ultrapure water system fitted with a Millipak^®^ 40 filter unit.

#### Synthesis of propargyl Nile red **1**

2-Hydroxy Nile red [37] (140.1 mg, 0.41 mmol) was dissolved in anhydrous DMF (5 mL). The solution was cooled in an ice bath followed by the addition of potassium carbonate (210.7 mg, 1.5 mmol) and propargyl bromide (100 µL, 1.5 mmol). The mixture was heated at 70 °C and stirred for 3 h. After the removal of the solvent, the residue was purified by column chromatography (EtOAc/hexanes 1:4) to obtain **1** (108.1 mg, 70% yield) as a dark purple solid. ^1^H NMR (400 MHz, DMSO-*d*_6_) δ 1.17 (t, *J* = 6.9 Hz, 6H, C*H*_3_CH_2_), 3.51 (q, *J* = 6.9 Hz, 4H, CH_3_C*H*_2_), 3.67 (t, *J* = 2.2 Hz, 1H, ≡CH), 5.03 (d, *J* = 2.2 Hz, 2H, OCH_2_), 6.20 (s, 1H, ArH), 6.65 (d, *J* = 2.6 Hz, 1H, ArH), 6.83 (dd, *J* = 9.1 and 2.6 Hz, 1H, ArH), 7.32 (dd*, J* = 8.7 and 2.5 Hz, 1H, ArH), 7.64 (d, *J* = 9.1 Hz, 1H, ArH), 8.04 (d, *J* = 2.5 Hz, 1H, ArH), 8.08 (d, *J* = 8.7 Hz, 2H, ArH); ^13^C NMR (100 MHz, DMSO-*d*_6_) δ 181.3, 159.8, 151.8, 150.9, 146.5, 138.1, 133.5, 131.0, 127.2, 125.5, 124.0, 118.2, 110.1, 107.0, 104.1, 96.0, 78.8, 78.7, 55.9, 44.4, 12.4; IR (ATR) ν_max_: 3281.7, 3190.3, 2968.3, 2920.4, 2846.4, 1675.4, 1584.0, 1405.6 cm^−1^; HRMS (ESI–TOF): [M + H]^+^ calcd for C_23_H_21_N_2_O_3_, 373.1552; found, 373.1581; UV (MeOH) λ_max_ (ε): 553 (3.2 × 10^4^).

#### Synthesis of Nile red-modified acpcPNA

The 3-aminopyrrolidine-4-carboxylic acid (apc) modified acpcPNA was manually synthesized at a 1.5 µmol scale on Tentagel S-RAM resin (Fluka, 0.24 mmol/g) from the four Fmoc-protected pyrrolidinyl PNA monomers (A^Bz^, T, C^Bz^, G^Ibu^) and spacers [Fmoc-(1*S*,2*S*)-2-aminocycolpentanecarboxylic acid or (3*R*,4*S*)-3-(Fmoc-amino)-1-trifluoroacetylpyrrolidine-4-carboxylic acid] [43] according to the previously published protocol [26–27,31]. Lysine was included at both C- and N-termini to improve the water solubility. After the completion of the synthesis, the N-terminal Fmoc group was removed and the free amino group was capped by acetylation. The acpcPNA on the solid support was spilt to 0.5 µmol portions for a further labeling experiment and treated with 1:1 dioxane/aqueous NH_3_ at 60 °C overnight to remove the nucleobase- and apc-protecting groups. Fully deprotected acpcPNA (0.5 µmol) was treated with 4-azidobutanal (15 µmol, 30 equiv) in the presence of NaBH_3_CN (30 µmol, 60 equiv) and HOAc (30 µmol, 60 equiv) in MeOH (200 µL) at room temperature overnight. After an exhaustive washing with MeOH, the azide-modified acpcPNA was reacted with **1** (7.5 µmol, 15 equiv) in the presence of tris[(benzyl-1*H*-1,2,3-triazol-4-yl)methyl]amine [44] (TBTA, 30 µmol, 60 equiv), tetrakis(acetonitrile) copper(I) hexafluorophosphate (15 µmol, 30 equiv) and (+)-sodium-L-ascorbate (60 µmol, 120 equiv) in 3:1 (v/v) DMSO/*t*-BuOH at room temperature overnight. After the reaction was completed, the labeled acpcPNA was cleaved from the solid support with trifluoroacetic acid (500 µL × 30 min × 3). After drying and washing with diethyl ether, the residue was purified by reversed-phase HPLC and characterized by MALDI–TOF mass spectrometry (Microflex, Bruker Daltonics; α-cyano-4-hydroxycinnamic acid matrix, positive linear ion mode).

#### Spectroscopic studies

Samples for melting temperature, UV–vis, and fluorescence studies were prepared in 10 mM sodium phosphate buffer (pH 7.0) at concentrations of PNA = 1.0 µM and DNA = 1.2 µM. UV–vis and thermal denaturation experiments were performed on a CARY 100 UV–vis spectrophotometer (Varian, Australia) equipped with a thermal melting system. Fluorescence spectra were measured on a Cary Eclipse Fluorescence Spectrophotometer (Varian, Australia) at an excitation wavelength of 580 nm with 5 nm excitation and emission slits. Fluorescence melting experiments were performed under the same conditions by measuring the sample that was pre-heated to the specified temperature at 5 °C intervals and left for equilibration at that temperature for at least 5 min. The emission at 643 nm was divided by the emission at 643 nm of the single-stranded acpcPNA at the same temperature and plotted against the temperature to obtain the fluorescence melting curves. Melting temperatures were obtained from the UV or fluorescence melting curves by first derivative plots.

## Supporting Information

File 1 NMR spectra, HPLC chromatogram, mass spectra and additional spectroscopic data.
