# Supplementary material for: Synthesis and optical properties of pyrrolidinyl peptide nucleic acid carrying a clicked Nile red label
Source: Beilstein J Org Chem. 2014 Sep 11;10:2166–74. doi: 10.3762/bjoc.10.224 (PMC4168962; doi:10.3762/bjoc.10.224)
Supplement: File 1 — NMR spectra, HPLC chromatogram, mass spectra and additional spectroscopic data. [file Beilstein_J_Org_Chem-10-2166-s001.pdf]

Supporting Information

for

Synthesis and optical properties of pyrrolidinyl peptide nucleic acid carrying a clicked Nile red label

Nattawut Yotapan[1], Chayan Charoenpakdee[1], Pawinee Wathanathavorn[1], Boonsong Ditmangklo[1], Hans-Achim Wagenknecht*[2] and Tirayut Vilaivan*[1]

Address: [1]Organic Synthesis Research Unit, Department of Chemistry, Faculty of Science, Chulalongkorn University, Phayathai Road, Patumwan, Bangkok 10330, Thailand and [2]Institute of Organic Chemistry, Karlsruhe Institute of Technology (KIT), Fritz-Haber-Weg 6, 76131 Karlsruhe, Germany

Email: Tirayut Vilaivan - vtirayut@chula.ac.th; Hans-Achim Wagenknecht - wagenknecht@kit.edu

*Corresponding author

NMR spectra, HPLC chromatogram, mass spectra and additional spectroscopic data

**Content**	**Page**
**Figure S1:** (a) ^{1}H NMR and (b) ^{13}C NMR spectra of propargyl nile red **1**	S3
**Figure S2:** HPLC chromatogram and MALDI-TOF mass spectrum of **10mer-Nr**	S4
**Figure S3:** HPLC chromatogram and MALDI-TOF mass spectrum of **11merAA-Nr**	S5
**Figure S4:** HPLC chromatogram and MALDI-TOF mass spectrum of **11merCC-Nr**	S6
**Figure S5:** HPLC chromatogram and MALDI-TOF mass spectrum of **11merGG-Nr**	S7
**Figure S6:** HPLC chromatogram and MALDI-TOF mass spectrum of **11merTT-Nr**	S8
**Figure S7:** UV-vis and fluorescence spectra of **1** in MeCN-buffer	S9
**Figure S8:** UV-vis and fluorescence spectra of **10mer-Nr** in MeCN-buffer	S9
**Figure S9:** UV-vis and fluorescence spectra of **10mer-Nr** with various mismatched DNA	S10
**Figure S10:** UV-vis and fluorescence spectra of **10mer-Nr** with various base-inserted DNA	S10
**Figure S11:** UV-vis and fluorescence spectra of **10mer-Nr** with various base-inserted DNA (indirect)	S11
**Figure S12:** UV-vis and fluorescence spectra of **10mer-Nr** with various base-inserted DNA (mismatched)	S11
**Figure S13:** UV-vis and fluorescence spectra of **11merAA-Nr** with complementary and base-inserted DNA	S12
**Figure S14:** UV-vis and fluorescence spectra of **11merCC-Nr** with complementary and base-inserted DNA	S12
**Figure S15:** UV-vis and fluorescence spectra of **11merGG-Nr** with complementary and base-inserted DNA	S13
**Figure S16:** UV-vis and fluorescence spectra of **11merTT-Nr** with complementary and base-inserted DNA	S13
**Figure S17:** UV-vis and fluorescence spectra of **10mer-Nr** and its DNA hybrids before and after addition of β-cyclodextrin	S14
**Figure S18:** Photographs of **11merXX-Nr** and its hybrids with various DNA under black light	S14

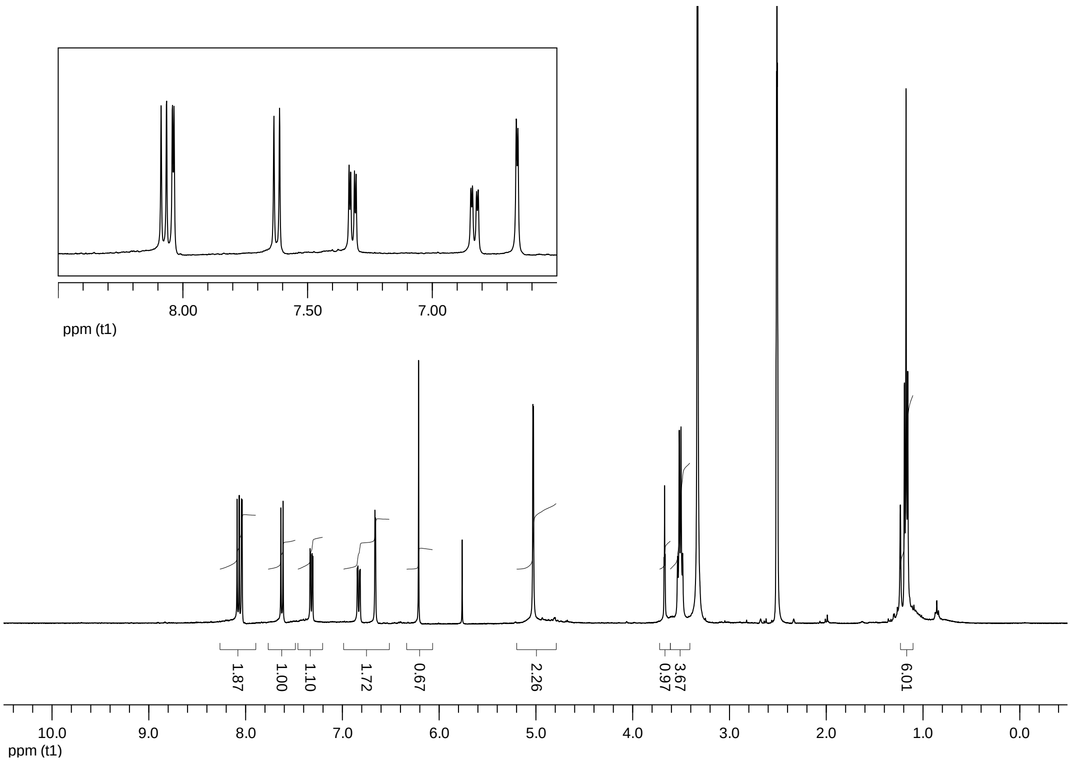

(a)

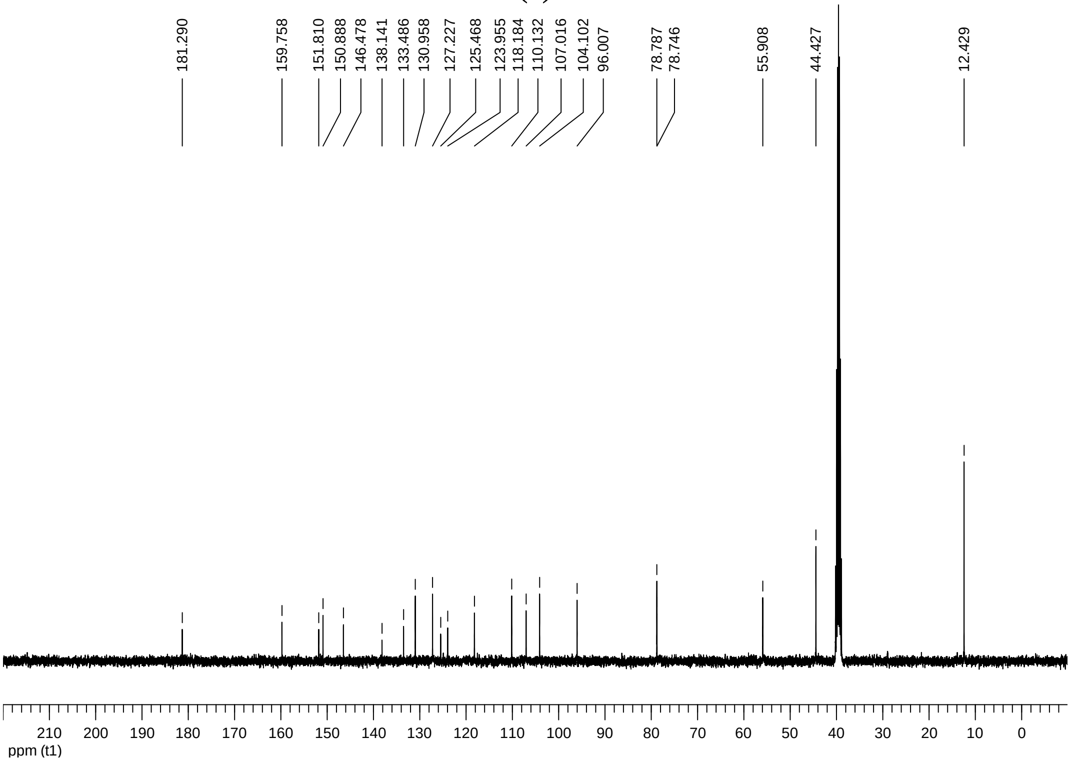

(b)

Figure S1: (a) ^{1}H NMR ($CDCl_3$, 400 MHz) and (b) ^{13}C NMR ($CDCl_3$, 100 MHz) spectra of propargyl nile red **1**

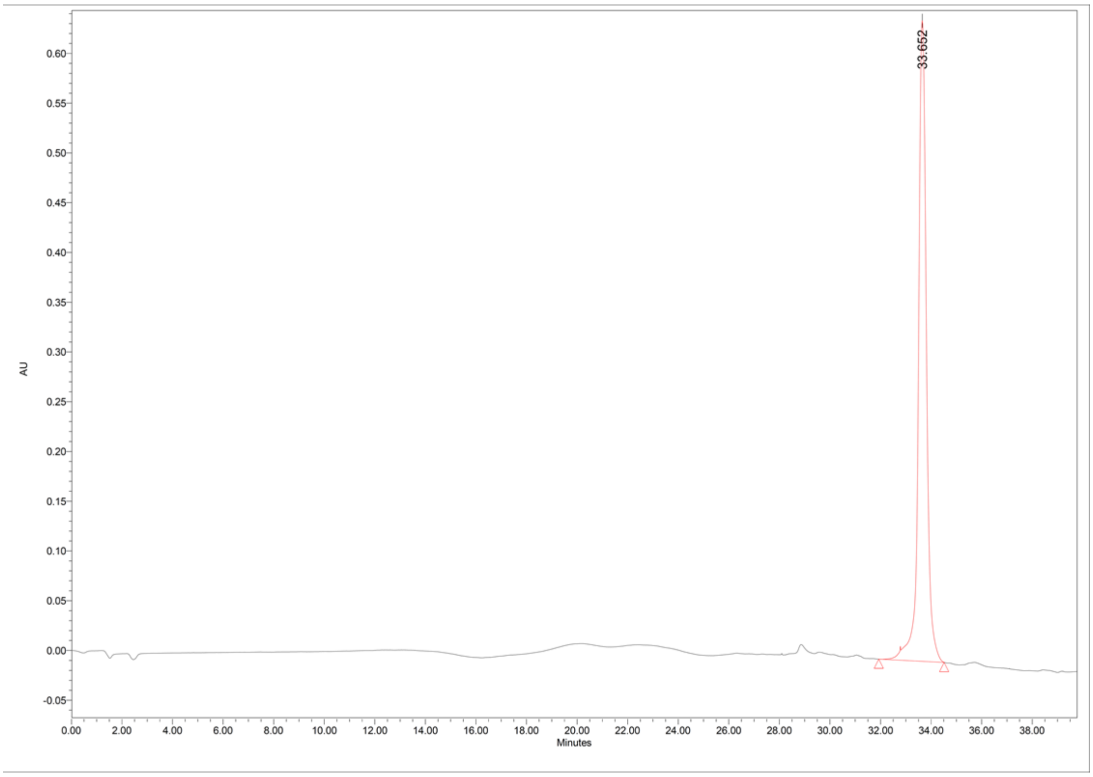

(a)

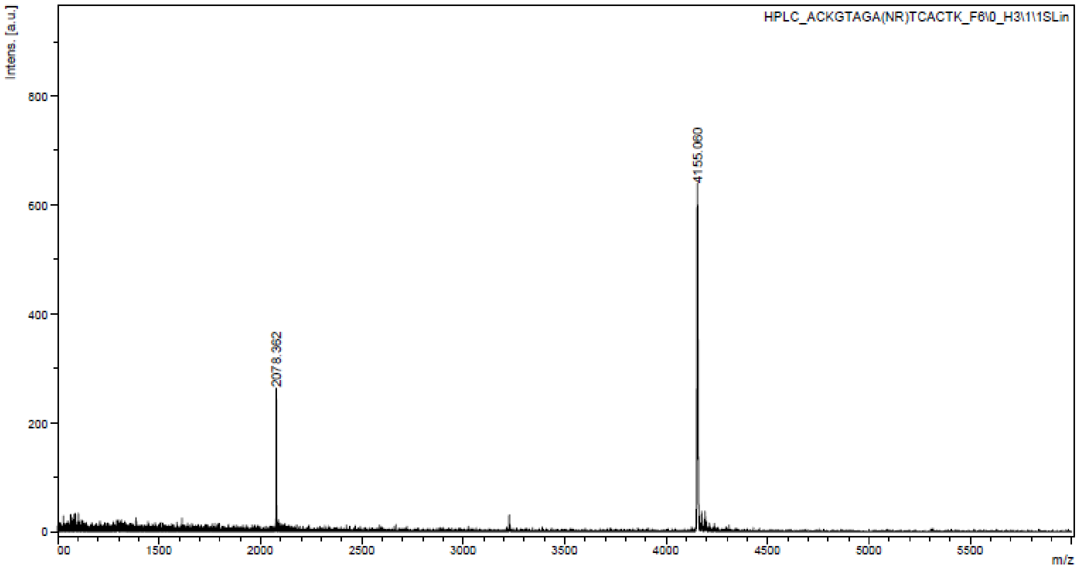

(b)

Figure S2: (a) Analytical HPLC chromatogram and (b) MALDI-TOF mass spectrum of **10mer-Nr** (calcd for $[M{\cdot}H]^+ = 4157.5$)

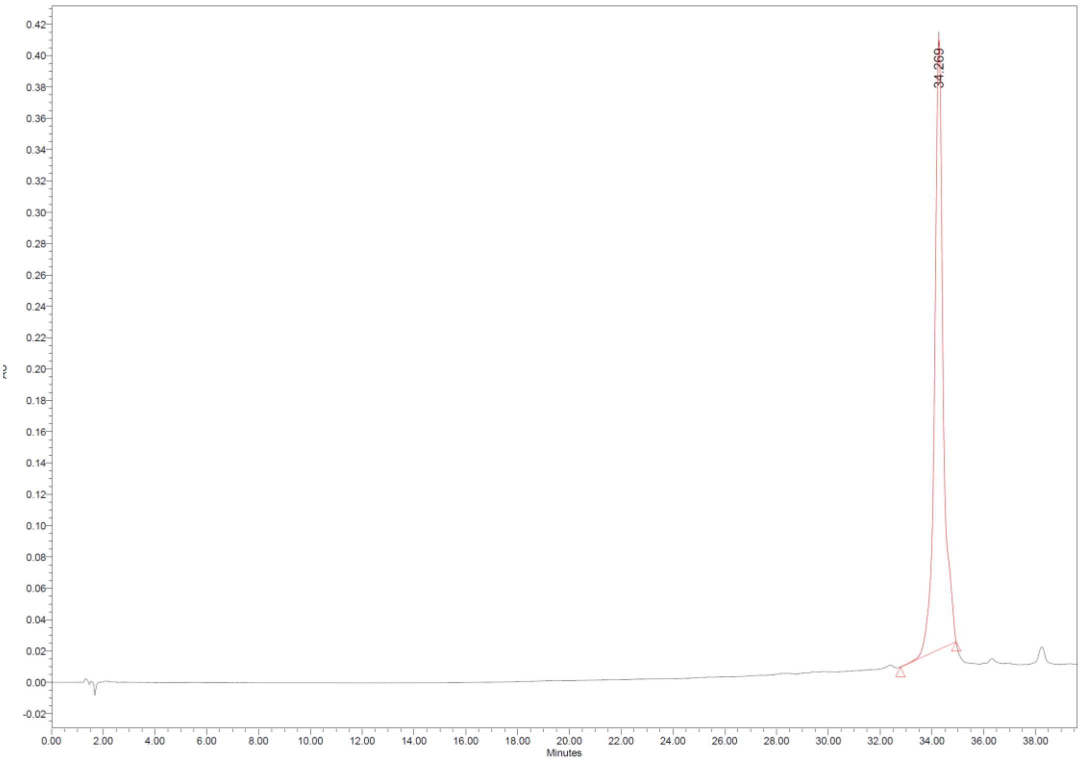

(a)

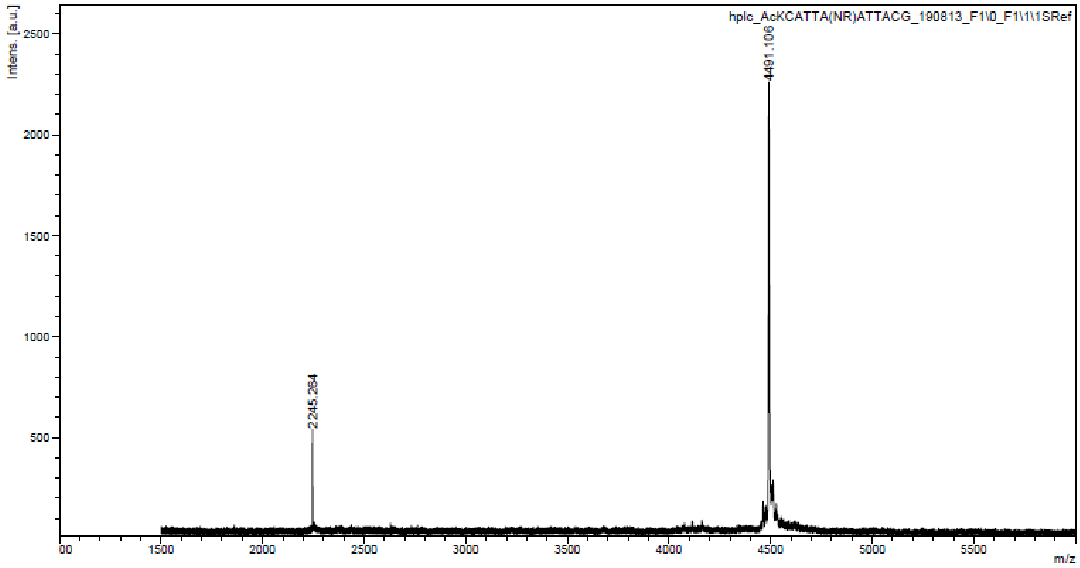

(b)

Figure S3: (a) Analytical HPLC chromatogram and (b) MALDI-TOF mass spectrum of **11merAA-Nr** (calcd for $[M{\cdot}H]^+$ = 4491.9)

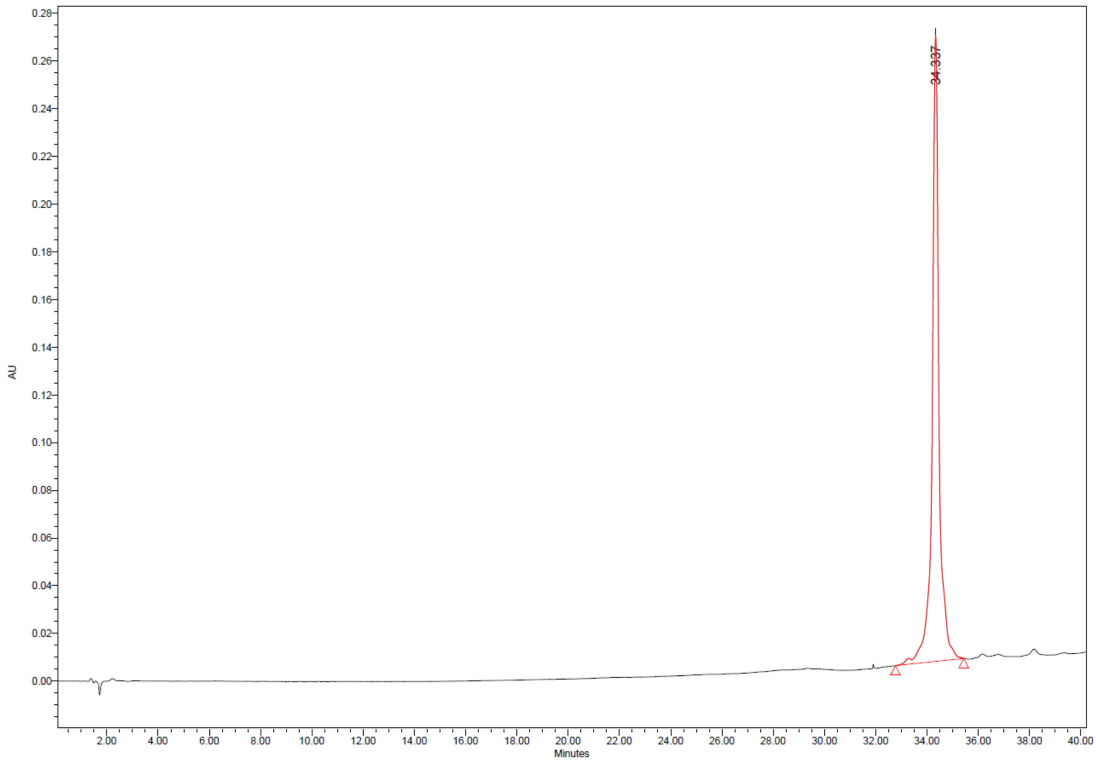

(a)

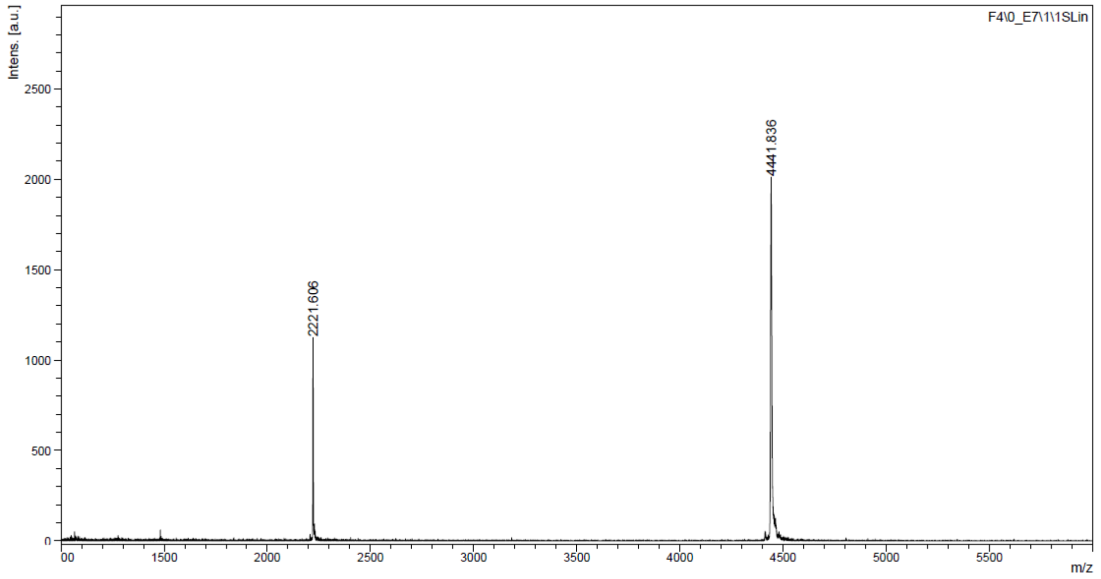

(b)

Figure S4: (a) Analytical HPLC chromatogram and (b) MALDI-TOF mass spectrum of **11merCC-Nr** (calcd for $[M{\cdot}H]^+ = 4443.9$)

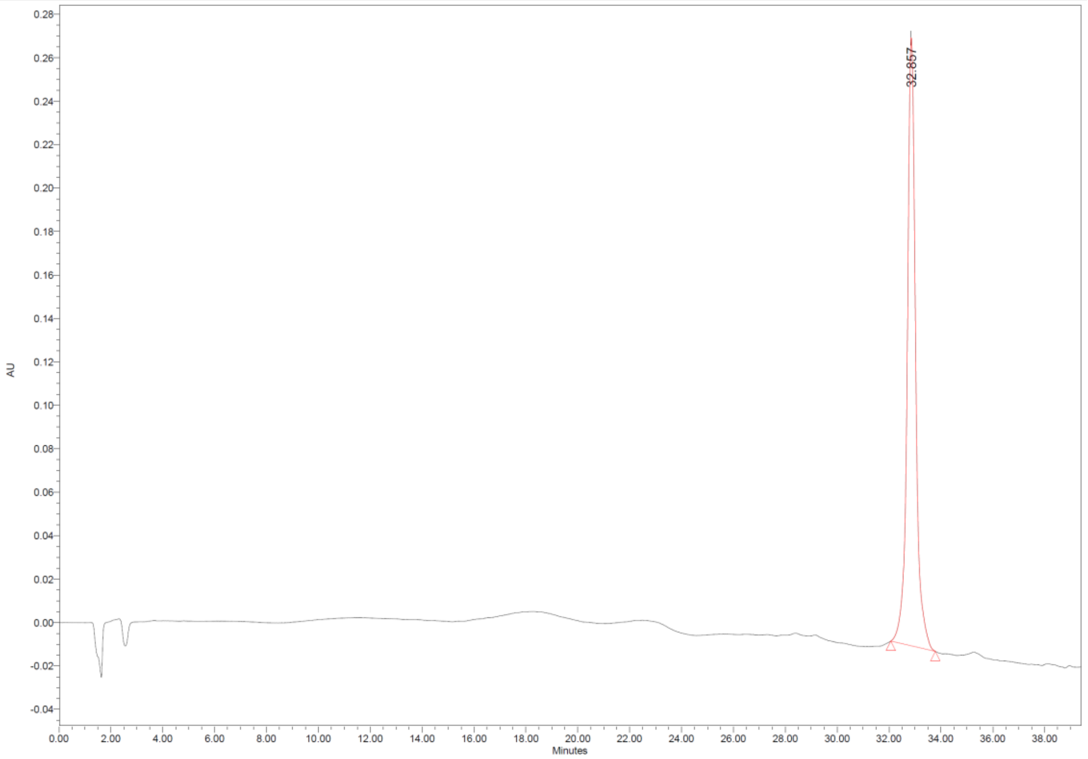

(a)

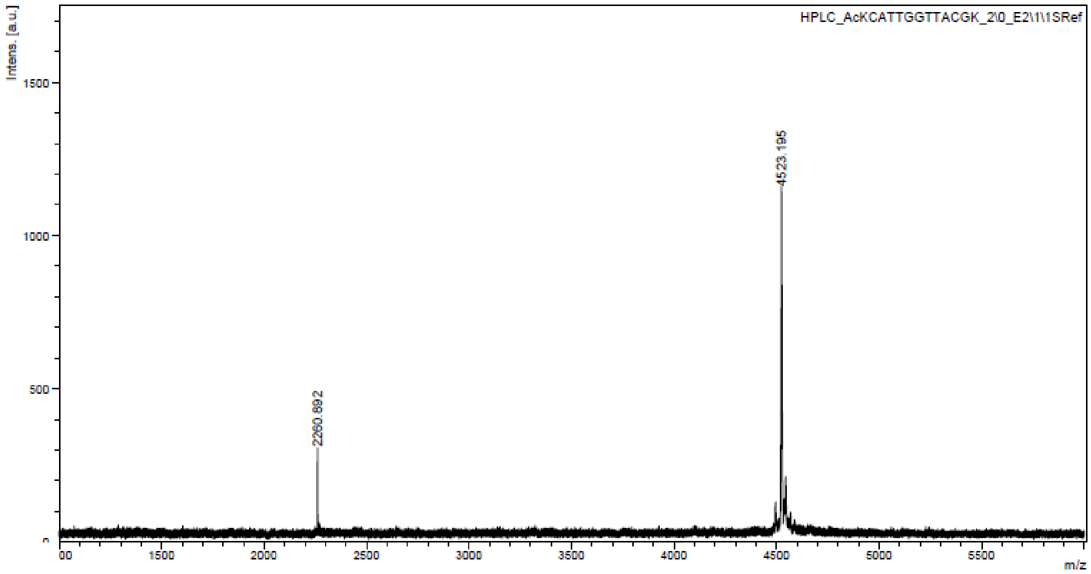

(b)

Figure S5: (a) Analytical HPLC chromatogram and (b) MALDI-TOF mass spectrum of **11merGG-Nr** (calcd for $[M{\cdot}H]^+$ = 4523.9)

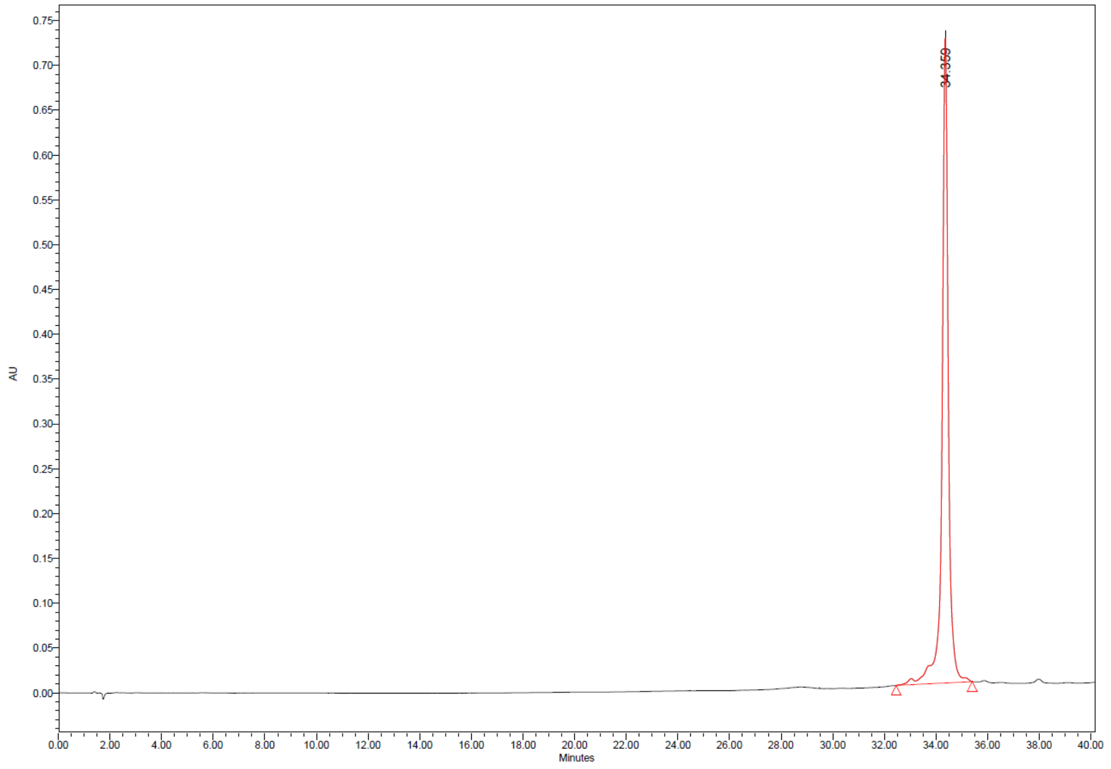

(a)

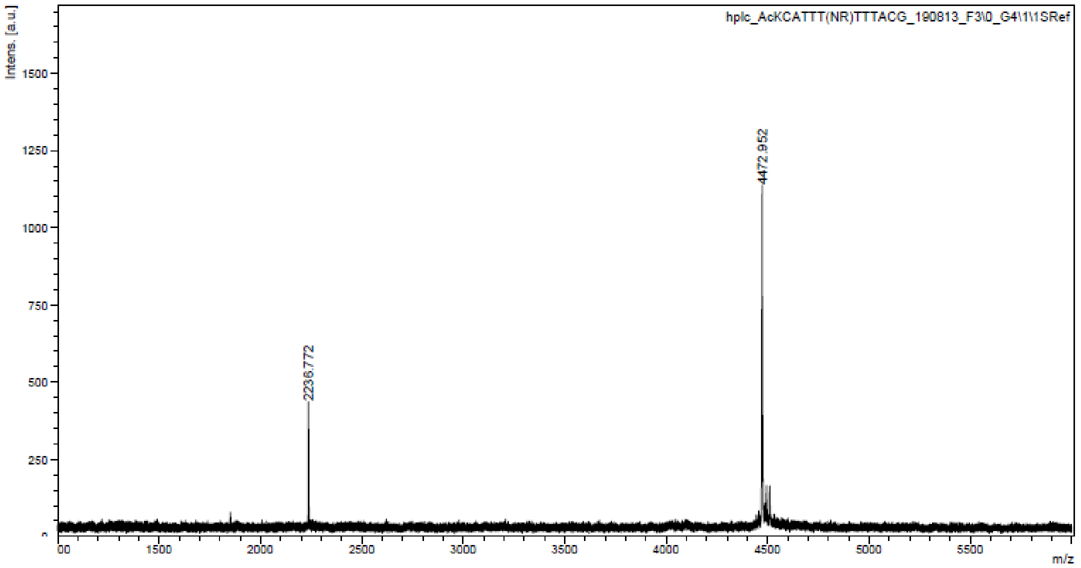

(b)

Figure S6: (a) Analytical HPLC chromatogram and (b) MALDI-TOF mass spectrum of **11merTT-Nr** (calcd for $[M{\cdot}H]^{+}$ = 4473.9)

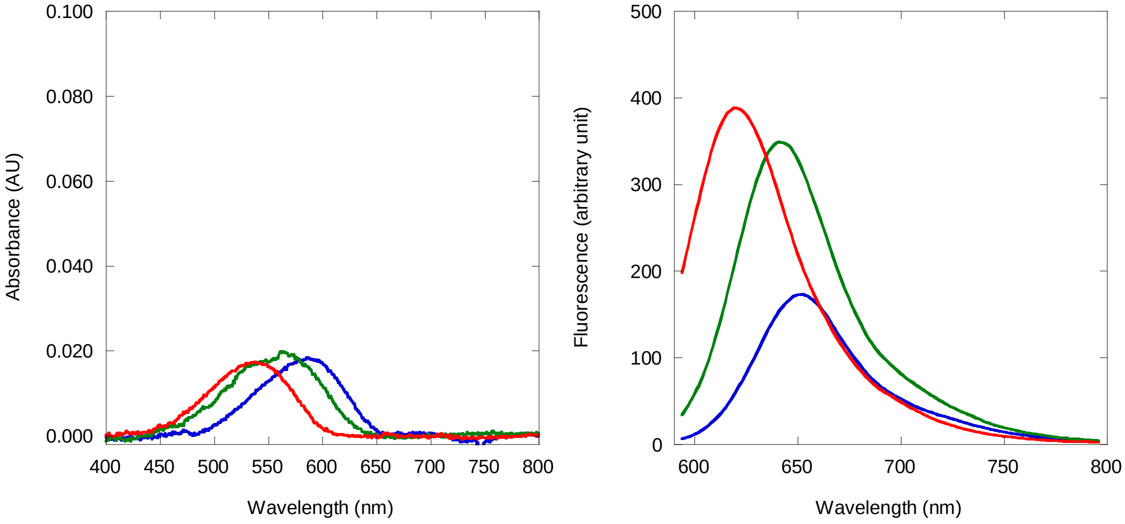

(a) (b)

Figure S7: (a) UV-vis and (b) fluorescence spectra of **1** in MeCN-buffer (10 mM sodium phosphate, pH 7.0): 20% MeCN (blue), 50% MeCN (green), 100% MeCN (red). All spectra were measured at [**1**] = 1.0 μM, λ_{ex} = 580 nm, 700 PMT.

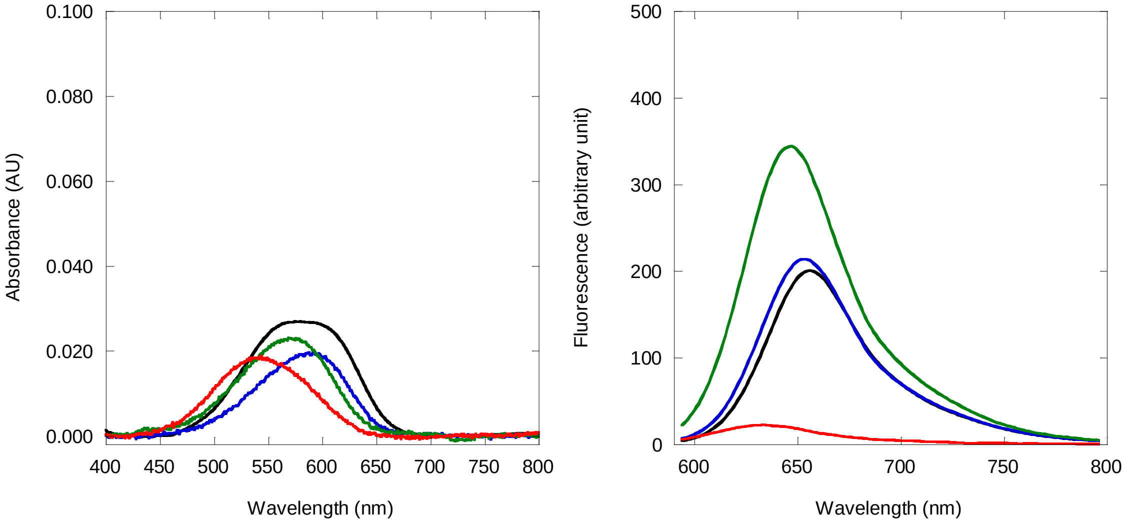

(a) (b)

Figure S8: (a) UV-vis and (b) fluorescence spectra of **10mer-Nr** in MeCN-buffer (10 mM sodium phosphate, pH 7.0): 0% MeCN (black), 20% MeCN (blue), 50% MeCN (green), 100% MeCN (red). All spectra were measured at [**1**] = 1.0 μM, λ_{ex} = 580 nm, 700 PMT.

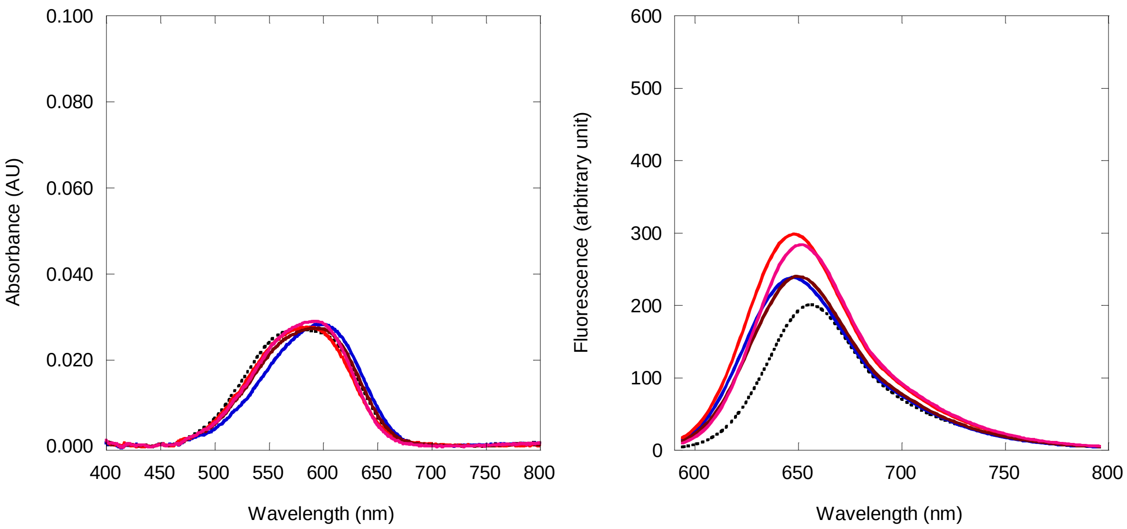

(a) (b)

Figure S9: (a) UV-vis and (b) fluorescence spectra of **10mer-Nr**: single stranded (black dotted), with 5'-d(AGTGATCTAC)-3' (blue), with 5'-d(AGTGCTCTAC)-3' (red), with 5'-d(AGTCATCTAC)-3' (brown), with 5'-d(AGTGACCTAC)-3' (pink). All spectra were measured in 10 mM sodium phosphate buffer pH 7.0, [PNA] = 1.0 μM, [DNA] = 1.2 μM, λ_{ex} = 580 nm, 700 PMT.

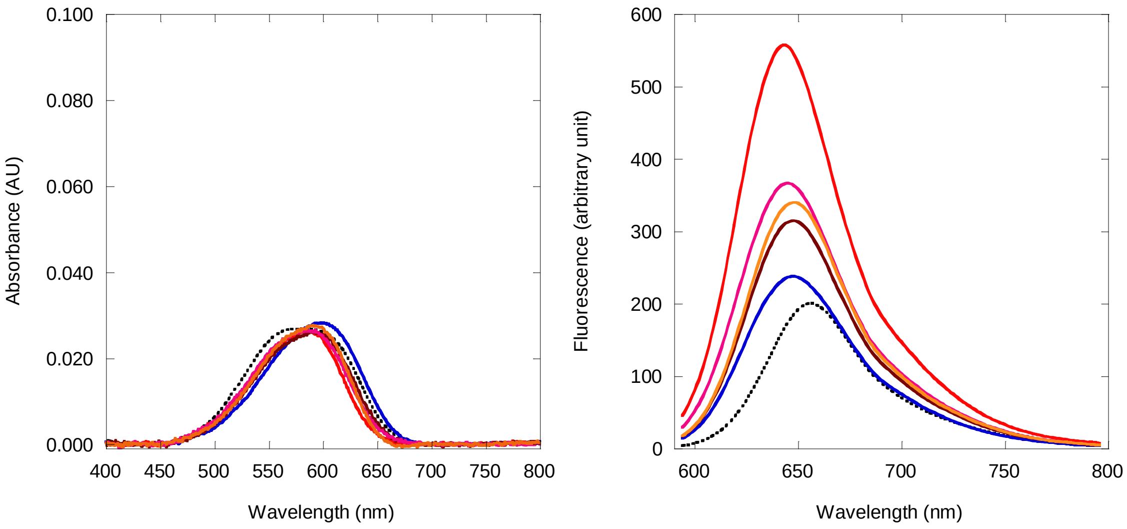

(a) (b)

Figure S10: (a) UV-vis and (b) fluorescence spectra of **10mer-Nr**: single stranded (black dotted), with 5'-d(AGTGATCTAC)-3' (blue), with 5'-d(AGTGA**C**TCTAC)-3' (red), with 5'-d(AGTGA**A**TCTAC)-3' (brown), with 5'-d(AGTGA**G**TCTAC)-3' (pink), with 5'-d(AGTGA**T**TCTAC)-3' (orange). All spectra were measured in 10 mM sodium phosphate buffer pH 7.0, [PNA] = 1.0 μM, [DNA] = 1.2 μM, λ_{ex} = 580 nm, 700 PMT.

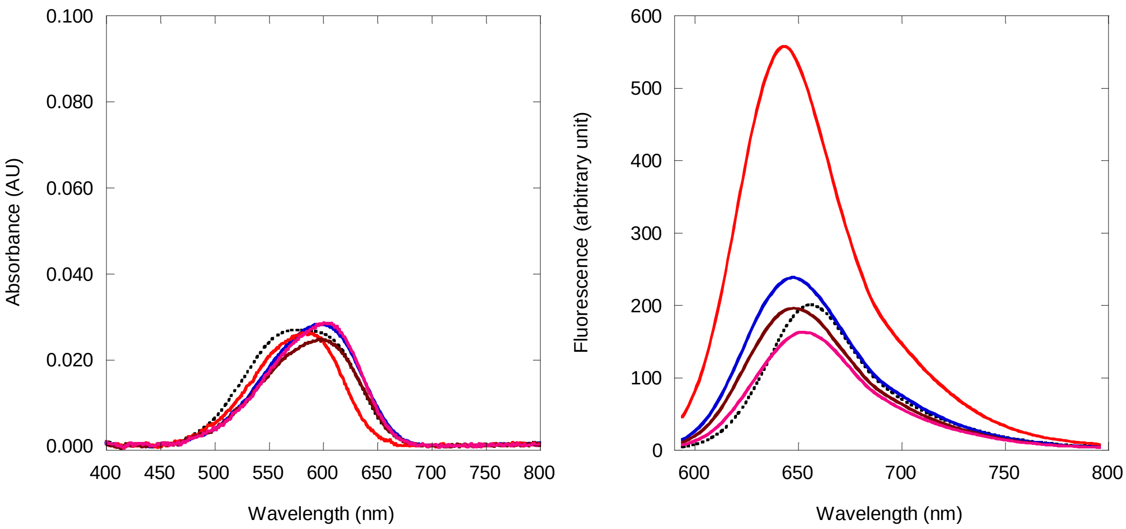

(a) (b)

Figure S11: (a) UV-vis and (b) fluorescence spectra of **10mer-Nr**: single stranded (black dotted), with 5'-d(AGTGATCTAC)-3' (blue), with 5'-d(AGTGA**C**TCTAC)-3' (red), with 5'-d(AGT**C**GATCTAC)-3' (brown), with 5'-d(AGTGATC**C**TAC)-3' (pink). All spectra were measured in 10 mM sodium phosphate buffer pH 7.0, [PNA] = 1.0 μM, [DNA] = 1.2 μM, λ_{ex} = 580 nm, 700 PMT.

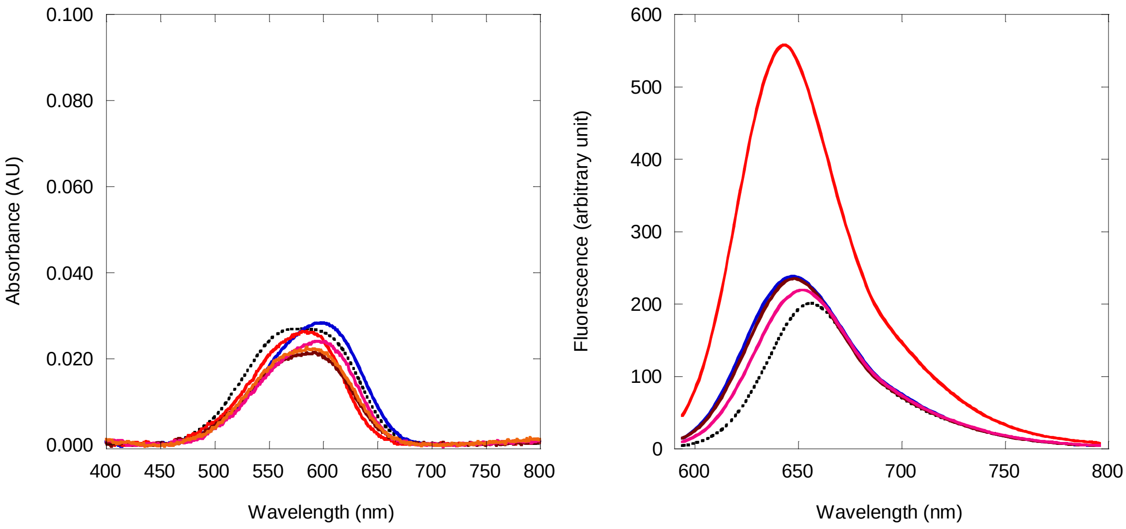

(a) (b)

Figure S12: (a) UV-vis and (b) fluorescence spectra of **10mer-Nr**: single stranded (black dotted), with 5'-d(AGTGATCTAC)-3' (blue), with 5'-d(AGTGA**C**TCTAC)-3' (red), with 5'-d(AGTGC**C**TCTAC)-3' (brown), with 5'-d(AGTGA**C**CCTAC)-3' (pink), with 5'-d(AGTGA**C**TCCAC)-3' (orange). All spectra were measured in 10 mM sodium phosphate buffer pH 7.0, [PNA] = 1.0 μM, [DNA] = 1.2 μM, λ_{ex} = 580 nm, 700 PMT.

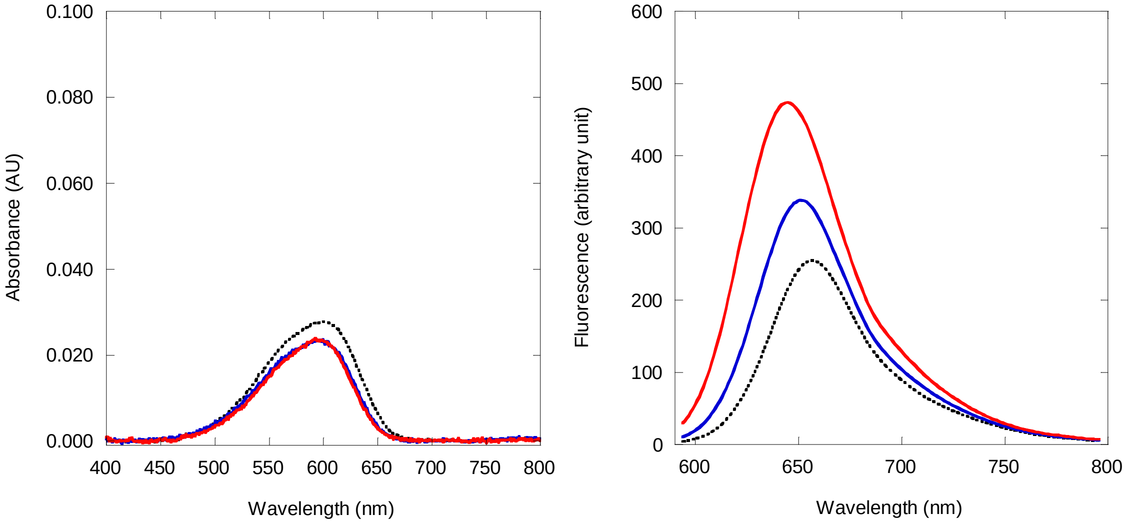

(a) (b)

Figure S13: (a) UV-vis and (b) fluorescence spectra of **11merAA-Nr**: single stranded (black dotted), with 5'-d(CGTATTTTATG)-3' (blue) and with 5'-d(CGTATT**C**TTATG)-3' (red). All spectra were measured in 10 mM sodium phosphate buffer pH 7.0, [PNA] = 1.0 μM, [DNA] = 1.2 μM, λ_{ex} = 580 nm, 700 PMT.

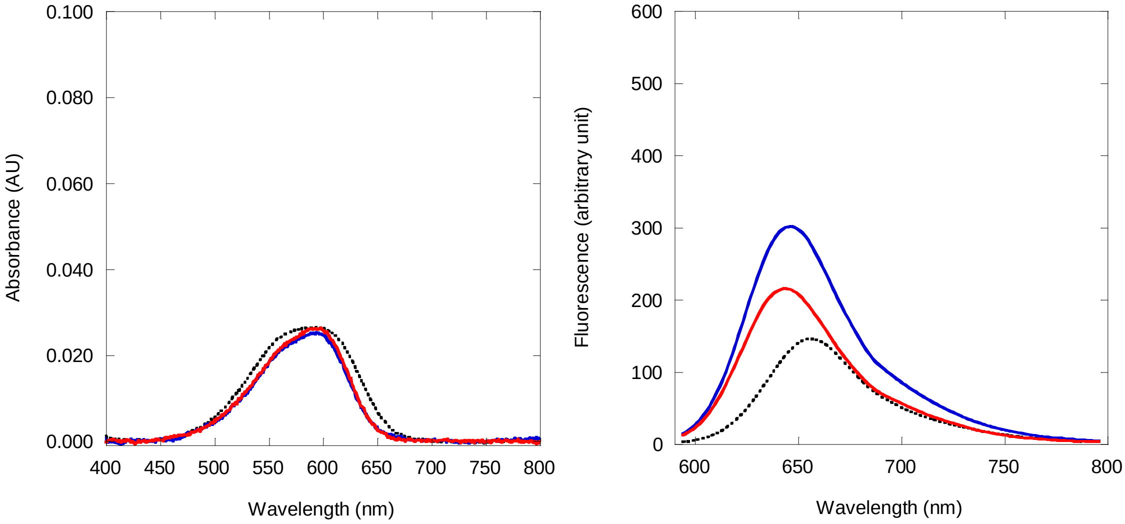

(a) (b)

Figure S14: (a) UV-vis and (b) fluorescence spectra of **11merCC-Nr**: single stranded (black dotted), with 5'-d(CGTATAATATG)-3' (blue) and with 5'-d(CGTATA**C**ATATG)-3' (red). All spectra were measured in 10 mM sodium phosphate buffer pH 7.0, [PNA] = 1.0 μM, [DNA] = 1.2 μM, λ_{ex} = 580 nm, 700 PMT.

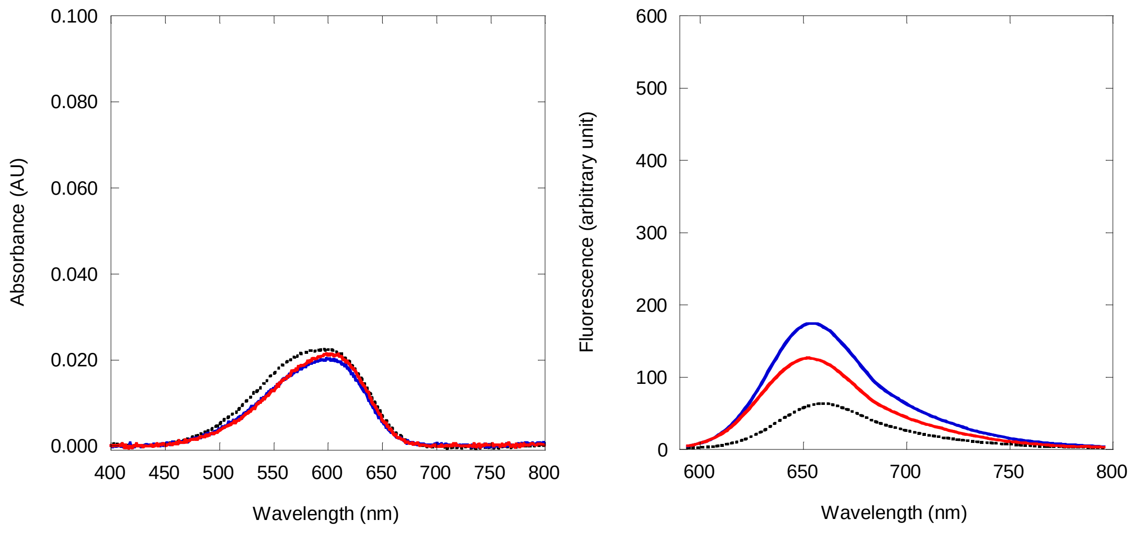

(a) (b)

Figure S15: (a) UV-vis and (b) fluorescence spectra of **11merGG-Nr**: single stranded (black dotted), with 5'-d(CGTATCCTATG)-3' (blue) and with 5'-d(CGTATC**C**CTATG)-3' (red). All spectra were measured in 10 mM sodium phosphate buffer pH 7.0, [PNA] = 1.0 μM, [DNA] = 1.2 μM, λ_{ex} = 580 nm, 700 PMT.

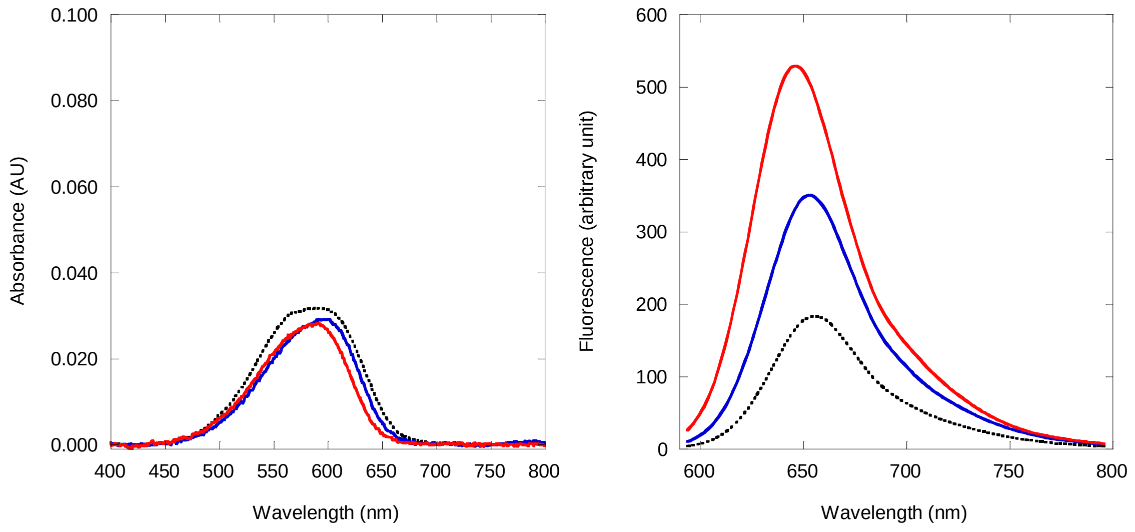

(a) (b)

Figure S16: (a) UV-vis and (b) fluorescence spectra of **11merTT-Nr**: single stranded (black dotted), with 5'-d(CGTATAATATG)-3' (blue) and with 5'-d(CGTATA**C**ATATG)-3' (red). All spectra were measured in 10 mM sodium phosphate buffer pH 7.0, [PNA] = 1.0 μM, [DNA] = 1.2 μM, λ_{ex} = 580 nm, 700 PMT.

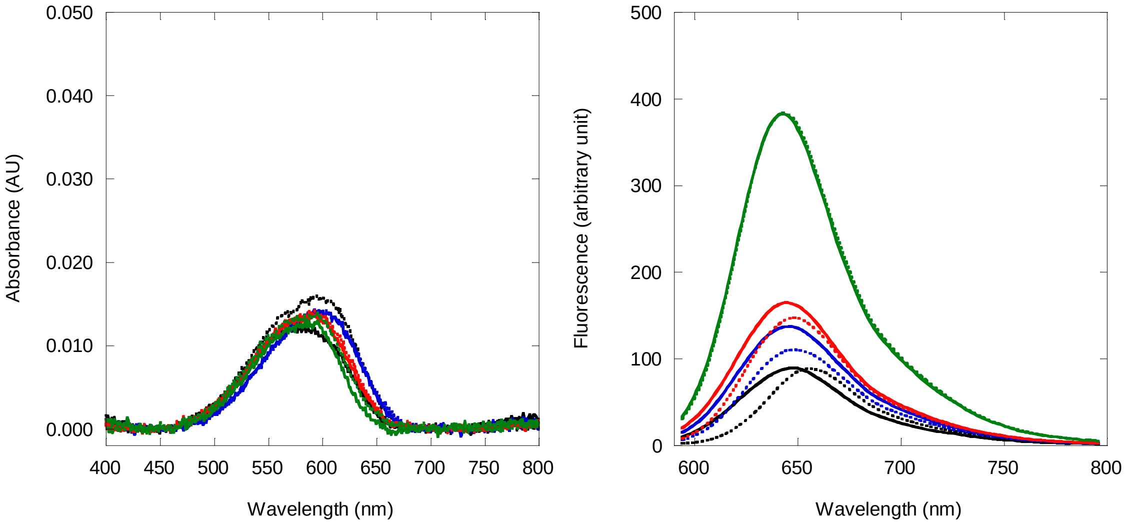

(a) (b)

Figure S17: (a) UV-vis and (b) fluorescence spectra of **10mer-Nr** and its DNA hybrids before (---) and after (—) addition of β-cyclodextrin (10 mM): single stranded (black), with 5'-d(AGTGATCTAC)-3' (blue),5'-d(AGTGCTCTAC)-3' (red), with 5'-d(AGTGA**C**TCTAC)-3' (green). All spectra were measured in 10 mM sodium phosphate buffer pH 7.0, [PNA] = 1.0 μM, [DNA] = 1.2 μM, λ_{ex} = 580 nm, 700 PMT.

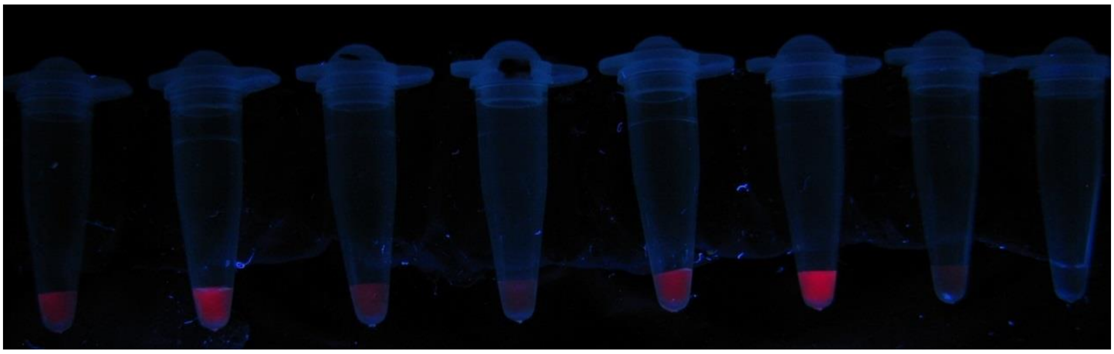

a **b** **c** **d** **e** **f** **g** **h**

Figure S18: Photographs of **11merXX-Nr** hybrids with DNA underblack light (405 nm): (a) **11merAA-Nr** and 5'-d(CGTATTTTATG)-3' (b) (a) **11merAA-Nr** and 5'-d(CGTATT**C**TTATG)-3' (c) **11merCC-Nr** and 5'-d(CGTATGGTATG)-3' (d) **11merCC-Nr** and 5'-d(CGTATG**C**GTATG)-3' (e) **11merTT-Nr** and 5'-d(CGTATAATATG)-3' (f) **11merTT-Nr** and 5'-d(CGTATA**C**ATATG)-3' (g) **11merGG-Nr** and 5'-d(CGTATCCTATG)-3' (h) **11merGG-Nr** and 5'-d(CGTATC**C**CTATG)-3'; Conditions: 10 mM phosphate buffer pH 7.0, [PNA] = 10 μM and [DNA] = 12 μM
